# Prospective therapeutics for intestinal and hepatic fibrosis

**DOI:** 10.1002/btm2.10579

**Published:** 2023-08-02

**Authors:** Xin Li, Mengli Yu, Qingwei Zhao, Yang Yu

**Affiliations:** ^1^ Department of Clinical Pharmacy, The First Affiliated Hospital Zhejiang University School of Medicine Hangzhou China; ^2^ Zhejiang Provincial Key Laboratory for Drug Evaluation and Clinical Research, The First Affiliated Hospital Zhejiang University School of Medicine Hangzhou China; ^3^ Institute of Pharmaceutics, College of Pharmaceutical Sciences Zhejiang University Hangzhou China; ^4^ Department of Gastroenterology, The Fourth Affiliated Hospital Zhejiang University School of Medicine Yiwu China; ^5^ College of Pharmaceutical Sciences Southwest University Chongqing China

**Keywords:** anti‐fibrotic strategies, challenges and prospects, hepatic fibrosis, intestinal fibrosis, nanotechnology

## Abstract

Currently, there are no effective therapies for intestinal and hepatic fibrosis representing a considerable unmet need. Breakthroughs in pathogenesis have accelerated the development of anti‐fibrotic therapeutics in recent years. Particularly, with the development of nanotechnology, the harsh environment of the gastrointestinal tract and inaccessible microenvironment of fibrotic lesions seem to be no longer considered a great barrier to the use of anti‐fibrotic drugs. In this review, we comprehensively summarize recent preclinical and clinical studies on intestinal and hepatic fibrosis. It is found that the targets for preclinical studies on intestinal fibrosis is varied, which could be divided into molecular, cellular, and tissues level, although little clinical trials are ongoing. Liver fibrosis clinical trials have focused on improving metabolic disorders, preventing the activation and proliferation of hepatic stellate cells, promoting the degradation of collagen, and reducing inflammation and cell death. At the preclinical stage, the therapeutic strategies have focused on drug targets and delivery systems. At last, promising remedies to the current challenges are based on multi‐modal synergistic and targeted delivery therapies through mesenchymal stem cells, nanotechnology, and gut‐liver axis providing useful insights into anti‐fibrotic strategies for clinical use.

**Translational Impact Statement**

The review describes the pathological mechanisms underlying intestinal and hepatic fibrosis, systematically summarizing and discussing anti‐fibrotic targets and the corresponding anti‐fibrotic strategies from the view of pathological mechanisms based on molecular, cellular, and tissue levels. The challenges and opportunities for developing anti‐fibrotic therapeutics are highlighted and discussed in detail. According to this, the potential opportunities and breakthroughs of anti‐fibrotic therapeutic methods are discussed, providing an incentive for clinical translation.

## 1 INTRODUCTION

Fibrosis is characterized by the excessive activation, proliferation, and differentiation of fibroblasts, the deposition of large amounts of extracellular matrix (ECM), and the destruction of tissue structure which can impair the function of almost any organ.^1^ Fibrosis has long been considered an irreversible disease, however, because of the progress in pathology studies and preventative therapeutic strategies, the reversal of fibrosis has come into focus in recent years.^2^, ^3^ The intestine and liver, are physiologically interconnected via the gut‐liver axis. The quality of life and even survival would declined for individuals following a diagnosis of intestinal or liver fibrosis. Although there are currently no effective therapies for intestinal or hepatic fibrosis, some therapeutic agents are currently being used in clinical trials for hepatic fibrosis at ClinicalTrials.gov. (Table 1). As the mechanisms underlying primary fibrosis are similar in different organs and include chronic inflammation and immune imbalance leading to the overactivation and proliferation of ECM‐producing cells, inspiration can be obtained from the latest anti‐fibrosis research.^4^ For example, Rurik et al. developed messenger RNA (mRNA)‐loaded lipid nanoparticles targeted to T cells in vivo to generate transient anti‐fibrotic chimeric antigen receptors, which were found to reduce fibrosis and restore cardiac function after injury.^5^ Long et al. developed a nanodecoy that mimicked fibroblasts using poly lactic‐co‐glycolic acid (PLGA) nanoparticles modified by an autologous skin fibroblast membrane. The nanodecoy carries a variety of cytokine receptors on its surface, which bind the corresponding fibrogenic cytokines.^6^ Another study used the peptide library carried by the phage to adhere to and isolate myofibroblasts in vivo. After amplification and screening in vitro, the polypeptide sequences targeting myofibroblasts were screened from cells. The targeted peptides enabled the precise delivery of sorafenib for anti‐renal fibrosis.^7^ Besides, several studies found a similarity between inflammatory bowel diseases (IBDs) and non‐alcoholic fatty liver disease (NAFLD), major causes of intestinal fibrosis and liver fibrosis respectively, in epidemiology across geographic areas over time, suggesting some close association between intestinal fibrosis and liver fibrosis.^8^ And this kind of association also indicated a therapeutic strategy on intestinal and hepatic fibrosis, which is discussed in Section 5.3. Here, this review will describe the pathological mechanisms underlying intestinal and hepatic fibrosis, systematically summarizing and discussing anti‐fibrotic targets and the corresponding anti‐fibrotic strategies from the view of pathological mechanisms based on molecular, cellular, and tissue levels. The challenges and opportunities for developing anti‐fibrotic therapeutics are highlighted and discussed. Lastly, the potential opportunities and breakthroughs of anti‐fibrotic therapeutic methods are discussed, providing an incentive for clinical translation.

**TABLE 1:** Examples of compounds in clinical phases II or III of development aimed at reducing hepatic fibrosis.

Target	Therapeutics	Modality	Phase	Primary outcome	NCT identifier
Dual agonist of GLP‐1 and GCGR	Cotadutide	Biomacromolecules	IIb/III	No results posted.	NCT05364931
Dual agonist of PPARα and PPARδ	Elafibranor	Small‐molecule	III	The primary endpoint of NASH remission for patients with considerable liver fibrosis was not achieved when treated with Elafibranor at 120 mg/d for 72 weeks.	NCT02704403
FGF21 analogues	Pegbelfermin BMS‐986036	Biomacromolecules	II	Pegbelfermin was generally well tolerated in the advanced NASH population. However, it did not meet the primary endpoint (≥1 stage). Improvement in the NASH‐CRN fibrosis score was assessed via biopsy.	NCT03486912
SCD1 inhibitor	Aramchol	Small‐molecule	III	A histological improvement in fibrosis (≥1 stage) was demonstrated for 39% of the patients according to NASH‐CRN.	NCT02279524
THR‐ β agonist	Resmetirom MGL‐3196	Small‐molecule	III	Resmetirom achieved both primary and critical secondary endpoints in phase III clinical trials (MAESTRO‐NASH) with good tolerance. The NAFLD activity score decreased by more than two points and was markedly higher than that in the placebo group.	NCT03900429
PNPLA3 gene inhibitor	AZD2693	Oligonucleotides	II	No results posted.	NCT05809934
CBP/β‐catenin inhibitor	PRI‐724	Small‐molecule	I/II	One out of every three patients who received the recommended PRI‐724 suffered a serious adverse reaction possibly related to PRI‐724. In addition, hepatic fibrosis was not decreased with any statistical significance after 12 weeks.	NCT03620474
HSC proliferation inhibitor	Hydronidone	Small‐molecule	II	There was a markedly significant improvement in liver fibrosis scores in the hydronidone group with good tolerance compared to the placebo group at week 52. In addition, the best improvement in the Ishak score was with the 90 mg/tid (270 mg/day) dosage.	NCT02499562
HSP47 mRNA	BMS‐986263	siRNA‐loaded LNP	II	BMS‐986263 was well tolerated after 12 weeks and showed improvement in METAVIR and Ishak scores in patients with chronic hepatitis C‐related fibrosis progression.	NCT03420768
LOXL2 inhibitor	Simtuzumab	Antibody	II	There was no therapeutic effect in decreasing hepatic collagen content or hepatic venous pressure gradient in phase IIb trials for patients with bridging fibrosis or compensated cirrhosis associated with nonalcoholic steatohepatitis.	NCT01672866 NCT01672879
Cyclophilin B inhibitor	Rencofilstat	Small‐molecule	II	Rencofilstat achieved the primary endpoint in ALTITUDE‐NASH markedly improving liver function in patients with advanced NASH. In addition, Rencofilstat achieved all secondary endpoints.	NCT05402371
CCR2 and CCR5 inhibitor	Cenicriviroc	Small‐molecule	II	Cenicriviroc was well tolerated, and significantly improved fibrosis without worsening NASH after 1 year of treatment compared with the placebo.	NCT02217475
Pan‐caspase inhibitor	Emricasan IDN‐6556	Small‐molecule	II	There was no improvement in liver histology in patients with NASH fibrosis. Although treatment with Emricasan lowered serum ALT in the short‐term, it may have led to alternative mechanisms of cell death.	NCT02138253

Abbreviations: CCR2, C‐C chemokine receptor 2; CCR5, C‐C chemokine receptor 5; FGF21, fibroblast growth factor 21; GCGR, glucagon receptor; GLP1, glucagon‐like peptide‐1; HSC, hepatic stellate cell; HSP47, heat shock protein 47; LNP, lipid nanoparticle; LOXL2, lysyl oxidase‐like 2; NAFLD, nonalcoholic fatty liver disease; NASH, nonalcoholic steatohepatitis; PNPLA3, patatin‐like phospholipase domain containing 3; SCD1, stearoyl‐coA desaturase; THR, thyroid hormone receptors.

## 2 INTESTINAL FIBROSIS

Intestinal fibrosis is a complication following chronic intestinal inflammation leading to the thickening of the gut wall and lumen strictures and which ultimately results in functional loss.^9^, ^10^ IBD, especially Crohn's disease (CD), involves chronic inflammation of the gastrointestinal (GI) tract and is the main cause of intestinal fibrosis.^11^

### 2.1 Pathogenesis of intestinal fibrogenesis

**FIGURE 1:**
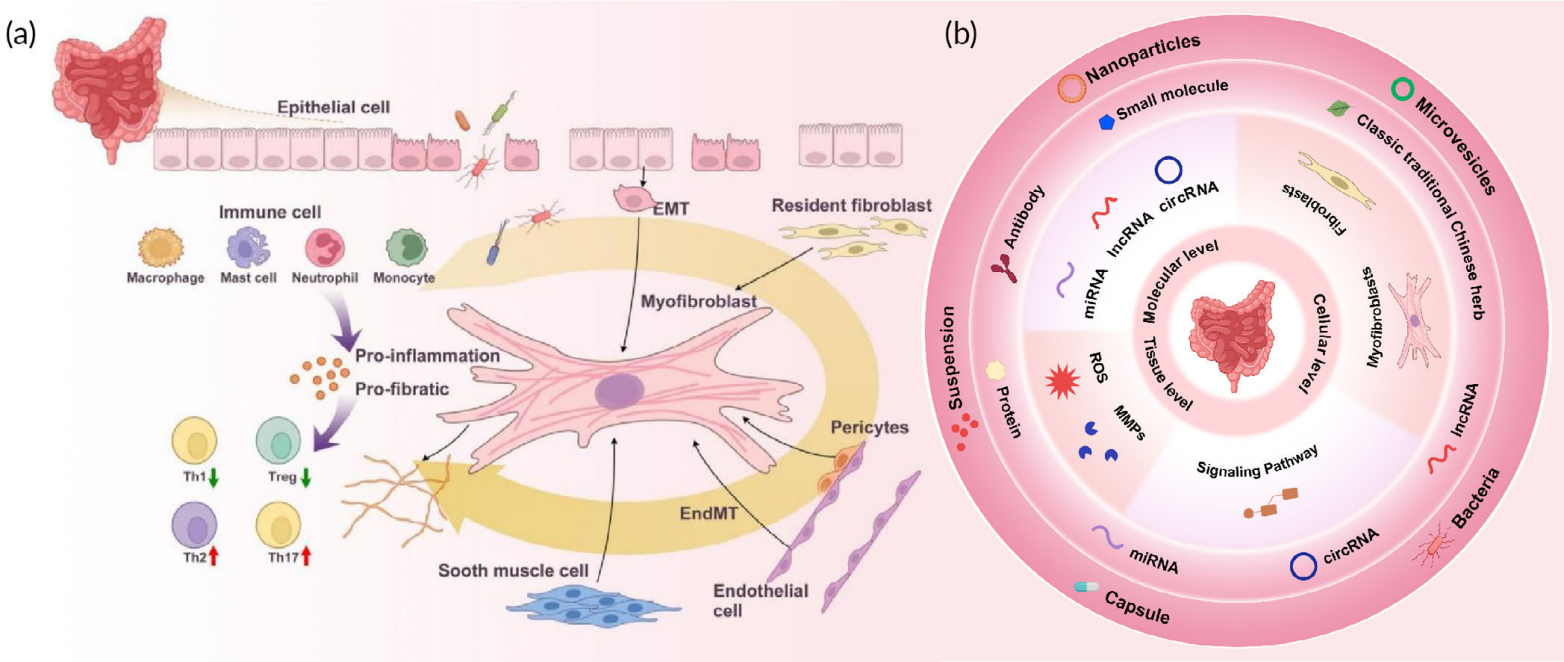
(a) Schematic illustration of the pathogenesis of intestinal fibrogenesis. As a result of chronic inflammation, the excessive immune response generates pro‐inflammatory and pro‐fibrotic molecules triggering the recruitment, proliferation, differentiation, and activation of ECM‐producing cells, which are central to the pathogenesis of fibrosis. The pro‐inflammatory and pro‐fibrotic factors released from the activated fibroblasts and immune cells produce a feedback loop, leading to an inflammation‐facilitated fibrosis process. (b) Anti‐fibrotic targets at different levels are summarized. Based on the understanding of the mechanisms underlying intestinal fibrogenesis, anti‐fibrotic targets at molecular, cellular, and tissue levels are summarized, and the corresponding therapeutic strategies are discussed.

Repeated intestinal injury activates innate and adaptive immune responses because of chronic inflammation.^12^ The excessive immune response generates pro‐inflammatory and pro‐fibrotic molecules triggering the recruitment, proliferation, differentiation, and activation of ECM‐producing cells, which are central to the pathogenesis of fibrosis^10^ (Figure 1a). ECM‐producing cells include myofibroblasts, smooth muscle cells, and pericytes, but myofibroblasts are the primary ECM‐producing cells. In addition to fibroblasts, epithelial and endothelial cells are important sources of myofibroblasts. Epithelial‐to‐mesenchymal transition (EMT) is a pathological process of cellular transdifferentiation in which epithelial cells acquire mesenchymal features, a fibroblast‐like profile, and show downregulated epithelial and upregulated mesenchymal markers.^13^ Endothelial‐to‐mesenchymal transition (EndMT) is also a pathological process of cellular transdifferentiation, however, in this case, the endothelium acquires mesenchymal features, a fibroblast‐like profile, and shows downregulated endothelial markers and upregulated mesenchymal markers.^10^ Both EMT and EndMT promote the development of intestinal fibrosis.^14^

### 2.2 Conventional therapeutic approaches

Since intestinal fibrosis is often associated with inflammation, traditional anti‐fibrotic therapeutic approaches depend on anti‐inflammatory agents. However, anti‐inflammatory therapies have demonstrated limited therapeutic effects on fibrosis. Corticosteroids may exert an indirect anti‐fibrotic effect beneficial for some fibrotic conditions by decreasing collagen synthesis and slowing the wound healing process.^15^ However, patients with fibrotic strictures often require surgery, and corticosteroids are associated with an increased risk of postoperative complications.^16^ Small‐molecule immunosuppressants may improve pulmonary function and reduce pulmonary fibrosis in patients with systemic sclerosis indicating that this class of drugs may prevent, or at least delay, intestinal fibrosis progression.^17^ However, there is no evidence to support the use of small‐molecule immunosuppressants, often used in the treatment of IBD, as an anti‐fibrotic drug in intestinal fibrosis.^18^ Studies investigating anti‐tumor necrosis factor α (TNF‐α) antibody effects on fibrosis have produced inconsistent results.^19^, ^20^ It is generally accepted that anti‐TNF‐α antibodies have no obvious therapeutic effect on intestinal fibrosis.

### 2.3 Targets and new therapeutic strategies

Although no drugs have been approved for the clinical treatment of intestinal fibrosis, an increasing number of studies are investigating therapeutic strategies. With the new drug delivery systems, the harsh GI tract environment is no longer a barrier for anti‐fibrotic drugs, particularly those developed from nucleic acid and biological macromolecules.^21^ Based on the current understanding of intestinal fibrogenesis, anti‐fibrotic targets, and the corresponding therapeutic strategies can be summarized at the molecular, cellular, and tissue levels (Figure 1b).

#### 2.3.1 Molecular level

Biomolecules, such as nucleic acids and proteins, are the basic functional units in cells that carry out important biological functions. Some aberrant events occur during intestinal fibrosis at the molecular level.^22^ In the following sub‐sections, these molecular level changes are summarized along with their corresponding therapeutics (Table 2).

**TABLE 2:** Targets and new therapeutic strategies for intestinal fibrosis.

	Targets	MoA	Therapeutics	Modality	Delivery system	References
Molecular level	Noncoding RNAs	Reduction of abnormal expression of colonic fibronectin collagen I protein	MiR‐200b	RNA	Microvesicles	^29^
	Signaling pathway	Inhibition of TGF‐β1 pathway	Vivomixx®	Multi‐strain probiotic	Capsule	^36^
		Inhibition of TGF‐β1 pathway	ACE‐I	Small‐molecule	PEG‐suspension	^37^
		Inhibition of Wnt/β‐catenin signaling	ICG‐001	Small‐molecule	N	^39^
		Inhibition of HIF pathway	BAH	Small‐molecule	N	^41^
		Inhibition of AXL pathway	BGB324	Small‐molecule	N	^42^
	Protein level	Competitive binding with IL‐36 receptors	Antibodies against IL36R	Antibody	N	^44^
		Induction of high tittered antibodies to IL‐12 and IL‐23	p40 peptide based vaccine	Peptide	N	^45^
		Reduction of IL‐13 and TGF‐β levels	pXYCYT:Hsp65	Bacteria	N	^46^
Cellular level	Myofibroblasts	Suppression on activation of myofibroblasts	Triptolide	Small‐molecule	N	^48^
		Disturbance of myofibroblast function	GED‐0507‐34 Levo	Small‐molecule	N	^49^
		Inhibition of myofibroblast accumulation and autophagy potentiation	AMA0825	Small‐molecule	N	^50^
		Inhibition of human colonic myofibroblast proliferation was reduced	Ang (1–7); Captopril	Small‐molecule	N	^51^
	Fibroblasts	Inhibition of fibroblasts proliferation	Pirfenidone	Small‐molecule	N	^53^
		Inhibition of accelerated fibroblast migration	Vitamin D	Small‐molecule	N	^54^
		Inhibition of fibroblast proliferation and activation	Wu‐Mei‐Wan	A traditional Chinese herb	N	^55^
Tissue level		Neutralization of excessive MMP‐9	Anti‐MMP‐9 antibody	Antibody	N	^59^
		Regulation of TIMP/MMP balance	Thalidomide	Small‐molecule	N	^60^
		ECM homeostasis reconstitution	Anti‐FAP antibody	Antibody	N	^61^
		Reduction in ROS levels	D‐CeO_2_ nanozyme	Small‐molecule	Dextran‐coated nanoparticle	^63^

Abbreviations: ACE‐I, angiotensin converting enzyme inhibitors; FAP, fibroblast activating protein; MMP, matrix metalloproteinase; MoA, mechanism of action; TIMP, tissue inhibitors of metalloproteinases.

##### Noncoding RNAs as anti‐fibrotic targets

Several studies have shown that noncoding RNAs (ncRNAs) present various modulatory functions in fibrogenesis. ncRNAs, such as microRNAs (miRNAs), long‐noncoding RNAs (lncRNAs), and circular RNAs (circRNAs), modulate fibrosis, and influence protein expression.^23^ The application of delivery systems has increased the feasibility of ncRNA manipulation in vivo making ncRNAs feasible therapeutic targets for various diseases.^24^, ^25^ miRNAs can be knocked down or complemented through the administration of antisense oligonucleotides or the delivery of miRNA mimics.^26^, ^27^ In IBD, various miRNAs are upregulated or downregulated at the intestinal fibrotic site compared to their expression in healthy tissues supporting the use of miRNA‐based therapeutics for intestinal fibrosis. The most widely known examples of miRNAs are the miRNA‐29 and miRNA‐200 families which are downregulated in the stricture mucosa of patients with CD.^28^ Increasing miRNA levels in the mucosa of lesions could be beneficial in intestinal fibrosis. Jia et al. developed miR‐200b‐loaded microvesicles (miR‐200b‐MVs) shown to protect miRNAs from degradation, transfer miRNAs to target cells, and attenuate colitis and intestinal fibrosis. Compared to a PBS (phosphate buffered saline)‐treated control, miR‐200b‐MVs reduced the expression of colonic fibronectin and collagen I by 28.08% and 51.65%, respectively, in rats with intestinal fibrosis.^29^ lncRNAs are long‐chain (>200 nucleotides) ncRNAs that play important roles in gene transcription, mRNA processing, and translation.^30^ The lncRNA HOX transcript antisense RNA (HOTAIR) has been shown to play a significant role in intestinal fibrosis through transforming growth factor (TGF‐β) signaling and EndMT modulation.^31^ CircRNA, a single‐stranded covalently closed circular RNA, is another subclass of ncRNAs. The functions of circRNAs are similar to those of lncRNAs and include modulating gene transcription and mRNA translation.^32^ It has been reported that Hsa_circRNA_102610 promotes TGF‐β1‐induced EMT by sponging miR‐130a‐3p in fibrosis.^33^ lncRNA and circRNA may also be prospective targets for intestinal anti‐fibrotic therapy, although this scenario has yet to be investigated. However, it is necessary to distinguish whether changes in ncRNA levels are causative or consequential. The same ncRNAs often modulate the expression of multiple gene targets creating the potential for off‐target effects.^34^, ^35^ When considering ncRNAs as a target for intestinal anti‐fibrotic treatment, the therapeutic agent, which is often also an ncRNA, must be able to enter the cell, and sometimes the nucleus, at sufficient concentrations which makes drug delivery a significant challenge.

##### Signaling pathways as anti‐fibrotic targets

**FIGURE 2:**
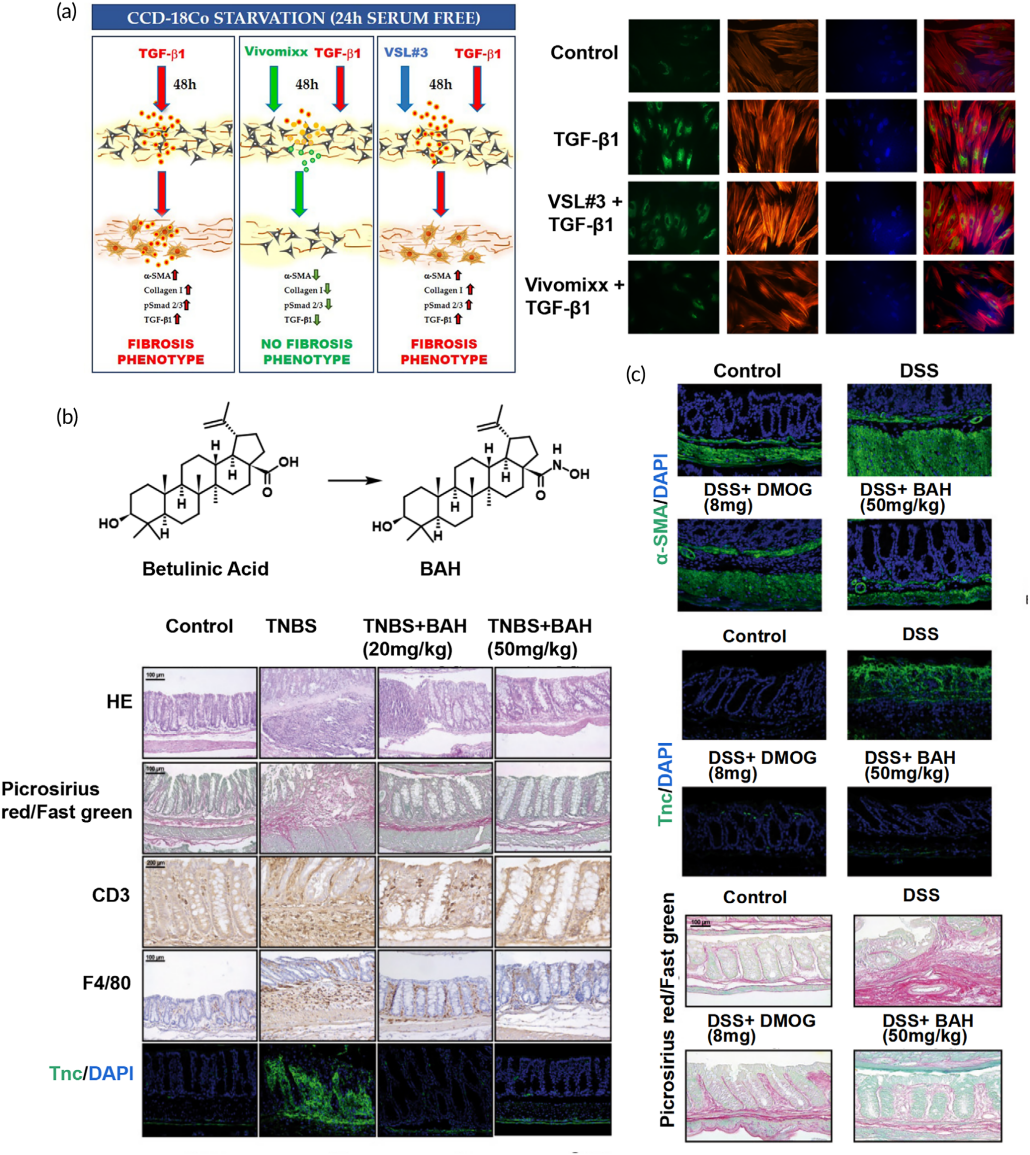
Strategies for anti‐intestinal fibrogenesis. (a) The lysates of multi‐strain probiotic formulations improve intestinal fibrosis in CCD‐18Co cells by inhibiting the TGF‐β1 pathway. Reproduced with permission from Reference 36, copyright 2021, Multidisciplinary Digital Publishing Institute. (b) and (c) Chemical structures of betulinic acid and betulinic acid hydroxamate (BAH); BAH attenuates fibrosis in the TNBS‐ and DSS‐induced fibrosis model. Reproduced with permission from Reference 41, copyright 2021, Springer Nature.

**FIGURE 3:**
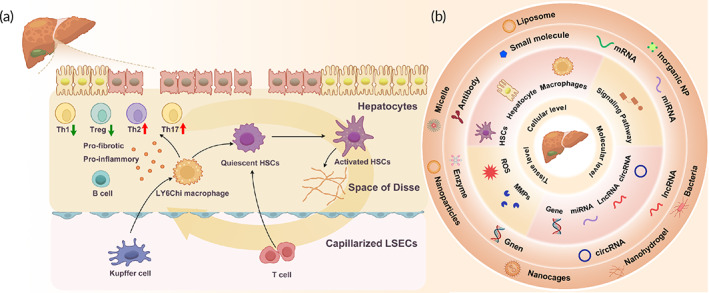
(a) Schematic illustration of the pathogenesis of hepatic fibrogenesis. In chronic liver disease, damaged hepatocytes, Kupffer cells, and macrophages release chemokines and cytokines to activate hepatic stellate cells (HSCs), which produce excessive extracellular matrix. The activated HSCs aggravate hepatocyte injuries and induce capillarization and accumulation of liver sinusoidal endothelial cells. The proinflammatory and pro‐fibrotic factors released from the HSCs and immune cells create a positive feedback loop. (b) Summary of anti‐fibrotic targets at different biological scales. Anti‐fibrotic targets at molecular, cellular, and tissue levels and their corresponding therapeutic strategies are summarized based on the understanding of the mechanisms underlying intestinal fibrogenesis.

Various signaling pathways participate in intestinal fibrosis development and several anti‐fibrotic targets have been identified. Vivomixx®, a mixed probiotic formulation, was found to prevent a fibrotic phenotype based on cellular and molecular parameters through inhibition of the TGF‐β1/Smad pathways (Figure 2a).^36^ Zhang et al. developed a chitosan‐modified PLGA nanoparticle for co‐delivering patchouli alcohol and simvastatin to alleviate colonic fibrosis by preventing the TGF‐β/Smad2/3 pathway. This nanoparticle effectively alleviated colonic fibrosis in a DSS‐induced intestinal fibrosis model.^37^ TGF‐β activates various pro‐fibrotic pathways in the intestine, however, it also possesses anti‐inflammatory activity and can regulate immunity through inducing Treg/Th17 cell differentiation, inhibiting dendritic cell proliferation, and promoting the anti‐inflammatory transformation of macrophages. Therefore, the complete elimination of TGF‐β is not required. Wnt/β‐catenin signaling was also demonstrated to be involved in the fibrotic process.^38^ Lewis et al. found that ICG‐001, an inhibitor of Wnt, could restrain fibrogenesis in CCD‐18Co cells and emphasized that cross‐talk between the Wnt and TGF‐β signaling pathways was involved in the pathogenesis of fibrogenesis^39^ making ICG‐001 a potential anti‐fibrotic therapeutic agent of multiple targets. It has been reported that hypoxic conditions are characteristic of IBD‐related fibrosis.^40^ Accordingly, betulinic acid hydroxamate (BAH), an inhibitor of hypoxia‐inducing factor (HIF) prolyl‐hydroxylases, was developed as a novel anti‐fibrotic therapy for IBD.^41^ BAH dose‐dependently activated the HIF pathway in both the 2,4,6‐trinitrobenzene sulfonic acid (TNBS)‐ and DSS‐induced fibrosis models. Continuous administration of BAH for 17 days significantly downregulated the expression of fibrotic markers, including collagen I, tissue inhibitors of metalloproteinases (TIMP‐1), and α‐smooth muscle actin (α‐SMA) (Figures 2b and 3c). Additionally, the AXL pathway was suggested to promote myofibroblast activation. Bemcentinib (BGB324), a small‐molecule inhibitor of the AXL pathway, was found to block the fibrotic process and prevent matrix‐stiffness in human intestinal organoids promoting myofibroblast apoptosis^42^ suggesting inhibition of the AXL pathway as a candidate for anti‐intestinal fibrosis.

##### Ligands and receptors as anti‐fibrotic targets

Some ligands produce pro‐fibrotic effects through cell membrane receptors that could be considered anti‐fibrotic targets. Concerning the important roles of interleukins (ILs) in activating and regulating immunity, some studies have found that ILs participate in intestinal fibrosis and many researchers are committed to developing anti‐fibrotic strategies against these targets. As a target for anti‐intestinal fibrosis, IL‐36 is the most widely studied.^43^ Scheibe et al. developed anti‐IL‐36 antibodies that bind competitively to IL‐36R and inhibit its downstream biological functions. Furthermore, they found that neutralizing antibodies against IL‐36R could reduce fibrosis and inflammation in the DSS‐ or TNBS‐induced fibrosis models suggesting that the inhibition of IL‐36 could be a strategy for anti‐intestinal fibrosis.^44^ Guan et al. showed that administration of a p40 peptide vaccine generated neutralizing antibodies against IL‐12 and IL‐23 producing an immunomodulatory effect by modifying the Treg/Th1 and Treg/Th17 ratios in the lamina propria to reduce TNBS‐induced intestinal fibrosis in mice.^45^ Furthermore, the level of the pro‐fibrotic cytokine IL‐13 could be regulated by the gut microbiota. Cunha et al. showed that oral administration of *Lactococcus lactis* NCDO2118 FnBPA+ (pXYCYT:Hsp65) reduced the levels of IL‐13 and attenuated the degree of fibrosis in mice with TNBS‐induced colitis.^46^

#### 2.3.2 Cellular level

Fibroblasts have long been recognized as an important source of myofibroblasts which are considered the chief effector cell in fibrogenesis.^47^ Considering the important roles of these cells in the occurrence and development of intestinal fibrosis, myofibroblast‐ and fibroblast‐based anti‐fibrotic strategies have been widely investigated (Table 2).

##### Myofibroblasts as anti‐fibrotic targets

Since activated myofibroblasts are the main ECM‐producing cells and represent the central mediators of intestinal fibrosis, strategies that prevent myofibroblast activation, block their function, or induce their death may decrease the production of ECM and improve fibrosis. Triptolide exerts anti‐inflammatory and immunomodulatory effects, however, its anti‐fibrotic effects remain unclear. In one study, a rat model of colonic fibrosis, induced by the administration of TNBS for 6 weeks, was treated with triptolide every day. PBS solution was used as the control. Triptolide inhibited the activation and suppressed the differentiation of myofibroblasts in rats with colonic fibrosis resulting in decreased ECM deposition in the colon.^48^ Speca et al. explored the anti‐fibrogenic efficiency of PPAR‐γ modulator agonists in the intestine and found that GED‐0507‐34 Levo disturbed myofibroblast function and suppressed fibrosis‐associated protein synthesis, suggesting that PPAR‐γ agonists may be a potential therapeutic drug candidate for intestinal fibrosis.^49^ Rho kinases (ROCKs) are highly activated in myofibroblasts. A study showed that prophylactic local administration of a ROCK inhibitor (AMA0825) prevented fibrogenesis in DSS‐ and adoptive T‐cell transfer‐induced fibrosis models. Therapeutic administration of AMA0825 reversed intestinal DSS‐induced fibrosis, likely because ROCK inhibition inhibited myofibroblast accumulation and potentiated autophagy.^50^ An increasing number of studies demonstrate that the renin‐angiotensin system (RAS) participates in regulating inflammation, fibrosis, and cell proliferation. In a study to assess the effects of RAS on intestinal fibrosis, the level of human colonic myofibroblast proliferation and collagen secretion was determined after treatment with angiotensin (Ang) II or Ang (1–7) solution.^51^ The results revealed that collagen secretion and the proliferation of human colonic myofibroblasts were reduced by Ang (1–7) and captopril demonstrating that therapeutic agents targeting RAS are very likely to improve fibrosis.

##### Fibroblasts as anti‐fibrotic targets

Strategies preventing fibroblast activation, accumulation, and transition into myofibroblasts would be beneficial for fibrosis treatment.^52^ In a study, pirfenidone (PFD) solution was added to fibroblasts isolated from biopsies taken from patients with CD. PFD inhibited the proliferation of the fibroblasts in the inflamed colonic mucosa suggesting that PFD may be applicable in intestinal fibrosis treatment.^53^ Gisbert‐Ferrándiz et al. found that vitamin D (VD) receptor expression decreased while fibroblast migration increased in intestinal stenosis tissue from patients with CD compared to healthy tissue. Following treatment with a VD solution, VD receptor levels increased preventing the accumulation of fibroblasts and the subsequent development of intestinal fibrosis induced by heterotopic transplant in mice, suggesting beneficial VD effects on intestinal fibrosis.^54^ In addition, Wu et al. found that the administration of Wu–Mei–Wan (WMW), a classic traditional Chinese herb medicine, could inhibit fibroblast proliferation and activation by decreasing ECM deposition and inhibiting TNBS‐induced intestinal fibrosis mice model compared to a placebo.^55^

#### 2.3.3 Tissue level

The tissue microenvironment has attracted increasing attention because it has unique features that are different from normal physiological conditions.^56^, ^57^ The microenvironment of intestinal fibrosis exhibits special characteristics which could be used to develop anti‐fibrotic therapies (Table 2). Matrix metalloproteinases (MMPs) are a group of proteases that degrade the ECM. Tissue inhibitors of metalloproteinase (TIMPs) are the primary endogenous MMP inhibitors. In normal intestinal tissue, the expression of MMPs and TIMPs is balanced, but in fibrosis this balance is disturbed contributing to collagen deposition. MMP‐9 is an MMP that can degrade ECM including gelatin and collagen.^58^ Goffin et al. found that the levels of the MMP‐9 degradation products were high in the serum of patients with CD and evaluated the effects of anti‐MMP‐9 antibodies in an intestinal fibrosis model. Anti‐MMP‐9 antibodies were found to neutralize excessive MMP‐9 reducing collagen deposition, suggesting that anti‐MMP‐9 antibodies may be an effective method for dampening fibrosis.^59^ Thalidomide, an immunomodulator, also has been demonstrated to inhibit intestinal fibrosis by regulating the TIMP/MMP protein balance and degradation of ECM.^60^ Truffi et al. further explored the differences between fibrotic and nonfibrotic mucosa in patients with CD and found that fibroblast activating protein (FAP) was upregulated in myofibroblasts from fibrotic mucosa compared to that from nonstenotic mucosa. Isolated bowel tissue cultures were treated with an anti‐FAP antibody revealing that the ECM in CD strictures was remodeled by TIMP‐1 expression inhibition and that reduced collagen production improved intestinal fibrosis.^61^ FAP expression is restricted to fibrotic areas and is not expressed in non‐strictured tissue making it suitable for identifying targets unique to pathological myofibroblasts. By targeting FAP on myofibroblasts, toxic drugs could be delivered to myofibroblasts, inducing their death and decreasing ECM production, without affecting normal intestinal tissues presenting new strategies for intestinal fibrosis treatment.

Metabolites such as the reactive oxygen species (ROS) have been demonstrated to play an important role in aggravating fibrosis. Evidence indicates that ROS activates and promotes TGF‐β signaling, in turn, TGF‐β1 boosts ROS production and decreases its elimination, which promotes fibrogenesis.^62^ Cao et al. developed a dextran‐coated cerium oxide (D‐CeO_2_) nanozyme by chemical precipitation which has potent ROS scavenging abilities to alleviate intestinal fibrosis. The D‐CeO_2_ nanozyme efficiently scavenged ROS and regulated the fibrosis‐related microenvironment in TNBS‐ and DSS‐induced intestinal fibrosis mice models, providing a practical use for nanozymes with ROS scavenging capabilities in anti‐fibrotic strategies.^63^

## 3 HEPATIC FIBROSIS

Hepatic fibrosis is characterized by excessive accumulation of the ECM and hepatocellular necrosis, followed by cirrhosis and hepatic failure which leads to approximately one million deaths annually.^1^ The pathogenesis of hepatic fibrosis includes viral infections, drug‐induced toxicity, metabolic disorders, cholestasis, and autoimmune disorders.^64^ Although there is currently no cure for hepatic fibrosis, some drugs have been effective at reducing symptoms, particularly those combined with nanoparticle delivery systems.

### 3.1 Pathogenesis of hepatic fibrogenesis

The effect of hepatic fibrosis during chronic liver disease involves inflammatory cytokines released by inflammatory cells which promotes the activation and proliferation of hepatic stellate cells (HSCs). HSCs are the primary abnormal ECM‐producing cells in the liver. Other ECM‐producing cells include fibroblasts and mesenchymal cells.^65^ HSCs, which account for 5%–8% of liver non‐parenchymal cells, are located in the subendothelial space between hepatocytes and sinusoidal endothelial cells, and are responsible for the storage of vitamin A and lipid droplets under healthy conditions.^66^ Upon liver injury, the HSCs are transformed from a quiescent condition to a myofibroblast‐like phenotype in two stages. First, chronic liver damage induces oxidative stress, resulting in the loss of lipid droplets from HSCs and their activation. Second, HSCs continuously release fibrogenic cytokines, and along with inflammatory cells, establish a pro‐fibrogenic environment at the tissue level (Figure 3a).^67^

### 3.2 Conventional therapeutic approaches

Therapeutic approaches for hepatic fibrosis include etiological and anti‐fibrotic treatments. Conventional treatment is aimed at treating the primary disease, such as antiviral drugs in viral hepatitis, anti‐inflammatory drugs in autoimmune liver disease, and antiparasitic drugs in schistosomiasis. Although the etiological treatment of liver fibrosis is fundamental, it does not prevent its progression and presents a limited therapeutic effect. Some studies have demonstrated that interferon‐γ (IFN‐γ) and IL‐10 possess anti‐hepatic fibrotic effects in vitro. However, the effects in vivo are limited.^68^ Chinese herbal medicines, such as Fuzheng huayu capsules, have also been used to treat anti‐hepatic fibrosis.^69^, ^70^ It is necessary to develop new therapeutic strategies with good efficacy and few side effects to treat hepatic fibrosis.

### 3.3 Targets and new therapeutic strategies

An increasing number of studies are investigating therapies for hepatic fibrosis, particularly combined with nano‐delivery technology. The application of the nanoparticle system is a unique advantage for liver disease since approximately 30%–99% of unmodified nanoscale particles preferentially accumulate in the liver following intravenous (IV) administration.^71^ Moreover, some receptors are overexpressed during the process of hepatic fibrosis. In the following sections, anti‐fibrotic targets are summarized at different levels and the corresponding therapeutic strategies are discussed (Figure 3b).

#### 3.3.1 Molecular level

Aberrant molecular events occur in genome transcription, translation, and protein metabolism in the occurrence and development of hepatic fibrosis.^72^ The extensive knowledge of these differential pathogenic molecular events has led to the identification of several promising therapeutic targets (Table 3).

**TABLE 3:** Targets and new therapeutic strategies for hepatic fibrosis.

	Targets	MoA	Therapeutics	Modality	Delivery systems	References
Molecular level	Gene	Silenced the HMGB1 gene	HMGB1‐siRNA	siRNA	NAL nanoparticles	^73^
		Regulation of pro‐fibrotic genes in HSCs	miRNA‐29b, miRNA‐122	miRNA	Micelle	^74^
	Noncoding RNAs	Knockdown of miR‐221‐3p	TuD‐miR‐221‐3p	miRNA	AAV8	^76^
		Dysregulated miR‐34a	Pterostilbene	Small‐molecule	N	^78^
		Increased circRNA SCAR expression located in the mitochondria	CircRNA SCAR expression vectors	pcD‐ciR	Mitochondria‐targeting NPs	^84^
	Signaling pathway	Inhibited TGF‐β1/Smad3 signaling	Umbelliferone	Small‐molecule	N	^85^
		Inhibited hedgehog signaling	Vismodegib	Small‐molecule	cRGDyK‐liposome	^87^
		Inhibited TGF‐β signaling	TGF‐βR blocker	Protein	*Lactococcus lactis*	^88^
Cellular level	Hepatocyte	Hepatocyte protection	Betulinic acid	Small‐molecule	Chitosan NPs	^93^
		Improved fibrotic primary hepatocyte functions	HNF4A mRNA	mRNA	Lipid NPs	^94^
	HSCs	Inhibited HSC activation	Imatinib	Small‐molecule	Liposomes	^96^
		HSC cytotoxicity activated	GMO, miR‐29b	Small‐molecule, miRNA	PEG‐PLGA NPs	^97^
		Inhibited HSC proliferation and activation	Quercetin	Small‐molecule	Nanocages	^98^
		Transformed aHSCs to quiescent phenotype	Relaxin‐plasmid, miR‐30a‐5p mimic	Gene, miRNA	LPH NPs	^99^
		Destroyed the Golgi structure and downregulated collagen I production	Retinoic acid, DOX	Small‐molecule	Micelles	^100^
		Broke down the dense collagen stroma and inhibited aHSC	Collagenase, silibinin	Enzyme, small‐molecule	NPs	^101^
		Thermal ablation	Gold	Inorganic material	Nanorods	^102^
	Hepatic immune cells	Macrophage repolarized toward an anti‐fibrotic M1 phenotype	CSF‐1R siRNA	siRNA	Nanohydrogel	^104^
		Modulated M2 macrophages	TSG‐6	Cytokine	CaP@BSA NPs	^105^
Tissue level		Reduced ROS levels	PD‐MC	Material	Micelle	^107^
		Reduced MMP‐9 mRNA expression	MMP‐9 siRNA	siRNA	Vitamin A‐liposome	^108^

Abbreviations: GMO, Germacrone; HMGB1, human high mobility group box‐1; HNF4A, hepatocyte nuclear factor 4 alpha; MMP, matrix metalloproteinase; MoA, mechanism of action.

##### Genes as anti‐fibrotic targets

**FIGURE 4:**
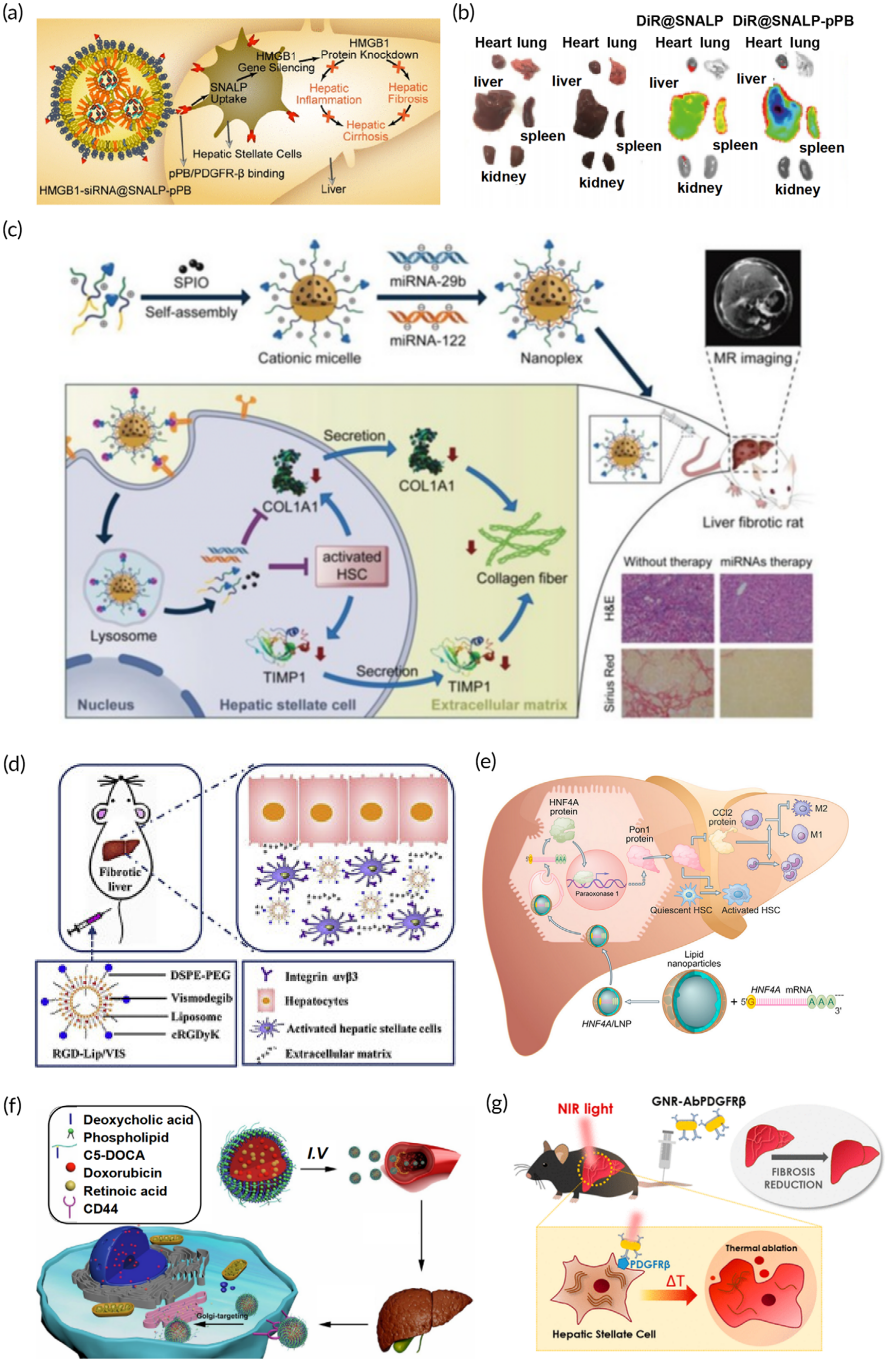
Strategies for anti‐hepatic fibrogenesis. (a) Human high mobility group box‐1‐siRNA@SNALP‐pPB effectively alleviate liver fibrosis. (b) The modification of pPB increases the accumulation of SNALP in the liver. Reproduced with permission from Reference 73, copyright 2020, American Chemical Society. (c) Schematic illustration of the miRNA loaded vitamin A‐modification of superparamagnetic iron oxide alleviating liver fibrosis. Reproduced with permission from Reference 74, copyright 2019, Wiley. (d) The Vismodegib loaded RGDLip/VIS reduced hepatic fibrosis by inhibiting hedgehog signaling in bile duct ligation and thioacetamide mice models. Reproduced with permission from Reference 87, copyright 2019, Elsevier. (e) Nanoparticles designed to deliver mRNA for recovery of hepatocytes. Reproduced with permission from Reference 94, copyright 2021, Elsevier. (f) The assembly of doxorubicin‐retinoic acid‐chondroitin sulfate micelles targeting the Golgi apparatus by *N*‐acetylgalactosaminyltransferase decoration. Reproduced with permission from Reference 100, copyright 2019, American Chemical Society. (g) The schematic illustration of gold nanorods‐PDGFRβ‐mediated photothermal therapy decreases carbon tetrachloride induced hepatic fibrosis in a mice model. Reproduced with permission from Reference 102, copyright 2021, American Chemical Society.

Genes are the most basic structures supporting life, storing all information about the process of life. Gene therapies have great potential in hepatic anti‐fibrotic treatment. The human high mobility group box‐1 (HMGB1) protein is a fibroblast chemokine that promotes hepatic inflammation and fibrosis. Zhang et al. developed HMGB1‐siRNA‐loaded lipid nanoparticles for the targeted silencing of the *HMGB1* gene in HSCs. The accumulation of lipid nanoparticles was markedly higher in liver tissue after modification with a pPB peptide. The activation and proliferation of HSCs was inhibited by the nanoparticles in vitro. Furthermore, this approach decreased collagen secretion in the liver and greatly increased the survival time in the TAA‐ and carbon tetrachloride (CCl4)‐induced hepatic fibrosis mouse model (Figure 4a,b).^73^ In another study, a cationic micelle assembled from a vitamin A (VA)‐conjugated copolymer was developed for the targeted delivery of miRNA‐29b and miRNA‐122 to HSCs. The results showed that the expression of fibrosis‐related genes was significantly downregulated by miRNA‐29b and miRNA‐122, improved hepatic function, and decreased the degree of fibrosis (Figure 4c).^74^

##### Noncoding RNAs as anti‐fibrotic targets

It has been reported that miRNA‐101 family members weaken TGF‐β signaling and miRNA‐223 inhibited fibrotic progression by targeting Cxcl10 and Taz in hepatocytes and macrophages.^75^ Researchers have found that miRNA‐221‐3p promoted liver fibrosis in the hepatocytes of mouse models, subsequently developing into AAV TuD‐miR‐221‐3p to downregulate its expression. The expression level of miR‐221‐3p decreased significantly in the AAV TUD‐miR‐221‐3p‐treated group compared to that in the control group. Additionally, fewer fibrotic areas and injuries in the liver were observed in mouse models of fibrosis.^76^ Dysregulation of miR‐34a has also been shown to be highly correlated with liver fibrosis.^77^ Pterostilbene has been demonstrated to downregulate miR‐34a expression, attenuating hepatocyte EMT, which may improve liver fibrosis.^78^ These results support the regulatory role of miRNAs in hepatic fibrosis. Moreover, some studies have shown that lncRNAs, such as H19, growth arrest‐specific transcript 5, Gm5091, and HIF 1 alpha‐antisense RNA 1 (HIF1A‐AS1) may inhibit the progression of liver fibrosis.^79^, ^80^ Thus, external supplementation of these lncRNAs provides a potential method to treat liver fibrosis. On the contrary, some lncRNAs have been demonstrated to promote liver fibrosis, including nuclear paraspeckle assembly transcript 1 (NEAT1), HOTAIR, and liver‐enriched fibrosis‐associated lncRNA1 (lnc‐LFAR1).^81^, ^82^, ^83^ The downregulation of overexpressed lncRNAs in patients could improve fibrosis. circRNA was also investigated in the treatment of liver fibrosis. Zhao et al. found that mitochondrial ROS production was inhibited by the mitochondrial steatohepatitis‐associated circRNA ATP5B regulator (SCAR), which prevented fibroblast activation. When circRNA SCAR expression vectors were constructed and encapsulated into mitochondria‐targeting nanoparticles, the nanoparticles alleviated the cirrhosis associated with a high‐fat diet in vivo, suggesting circRNAs as a novel target in the process of fibrosis in nonalcoholic steatohepatitis (NASH), and supporting the potential for circRNA as an anti‐fibrotic target.^84^ The use of ncRNAs as targets in liver fibrosis is challenging, but also promising.

##### Signaling pathways as anti‐fibrotic targets

Various fibrotic signaling pathways have been highlighted as important anti‐fibrotic targets in hepatic fibrosis. Umbelliferone (UMB), also known as 7‐hydroxycoumarin, demonstrated efficacy in the prevention of hepatic fibrogenesis in CCl4‐induced liver fibrosis by inhibiting TGF‐β1/Smad3 signaling and downregulating α‐SMA and collagen I expression.^85^ Letrozole has been demonstrated to ameliorate liver fibrosis by inhibiting the downstream pathway of CTGF.^86^ Vismodegib was loaded onto cRGDyK‐modified liposomes to inhibit the activation of HSCs by the inhibition of the hedgehog pathway. The cRGDyK‐modified liposomes showed affinity to αvβ3 on aHSC in vitro and in vivo, increased accumulation on aHSCs rather than on quiescent HSCs, and inhibited the progression of fibrosis in bile duct ligatonin thioacetamide mice models (Figure 4d).^87^ Bacteria have also been used as a “delivery tool” to produce therapeutic proteins. Yuan et al. constructed a recombinant *L. lactis* expressing the TGF‐βR extracellular domain bound to the TGF‐βR, which reduced hepatic fibrosis in CCl4‐treated mice. Recombinant bacteria with intrinsic biocompatibility represent a potential therapeutic strategy, particularly for the oral administration of biomacromolecules, reducing the degree of liver fibrosis.^88^ Because bacteria can produce and deliver target proteins, their use can markedly reduce the costs associated with drug production.^89^

#### 3.3.2 Cellular level

Hepatocytes, HSCs, and hepatic macrophages are key participants in the progression of whole liver fibrosis, and could be selected as therapeutic targets according to the following roles: (1) protecting hepatocytes from damage, reducing their apoptosis, and maintaining their function; (2) reversing the activated state of HSCs, inhibiting their proliferation, and promoting their apoptosis; and (3) promoting the transformation of hepatic macrophages to an anti‐fibrotic phenotype, and inhibiting the production of pro‐fibrotic factors (Table 3).^90^

##### Hepatocytes as targets

As hepatocyte necrosis is a primary mechanism underlying fibrosis, preventing hepatocyte damage via delivery of liver‐protecting drugs has potential in liver fibrosis treatment. Some receptors are highly expressed on hepatocytes during fibrogenesis and may be targeted for drug delivery.^91^ For example, the asialoglycoprotein receptor (ASGP‐R) has become a popular ligand for hepatocyte‐targeting, as it is highly expressed on the surface of hepatocytes in the process of fibrosis.^92^ Galactose, which specifically targets ASGP‐R, was modified on betulinic acid (BA)‐loaded chitosan nanoparticles to reduce the degree of liver injury in a liver fibrosis experimental model. BA is a pentacyclic triterpenoid acid that protects hepatocytes from damage. Galactosylated nanoparticles demonstrated increased drug accumulation in hepatocytes via galactose‐ASGP‐R interaction.^93^ ASGP‐R can also be viewed as an index to reflect the level of liver fibrosis giving galactose‐modified delivery systems potential in anti‐fibrotic therapies for diagnosis of the degree of liver fibrosis based on the accumulation of galactose in hepatocytes. In one study, Yang et al. reported the recovery of hepatocytes following hepatocyte nuclear factor 4 alpha (HNF4A) mRNA transfection in vitro. These authors developed a method of lipid nanoparticle‐mediated HNF4A mRNA delivery in hepatocytes which greatly inhibited fibrogenesis in models of liver fibrosis induced by hepatotoxin or cholestasis^94^ (Figure 4e).

##### HSCs as targets

Activated HSCs are the primary ECM‐producing cells in hepatic fibrosis and are regarded as central mediators of hepatic fibrosis. HSCs are also considered the primary target cells for many anti‐hepatic fibrosis strategies.^95^ Specific receptors and ligands expressed on the surface of HSCs during fibrogenesis could be targeted to increase delivery efficiency. Although the activation of HSCs was inhibited by imatinib in vitro, free imatinib therapy is hampered by low concentrations at the HSCs. Imatinib‐loaded liposomes modified with VA (ILC) were prepared, and when compared to free imatinib, the ILC presented strong anti‐fibrotic effects with reduced cytotoxicity.^96^ Germacrone (GMO) and miR‐29b were found to suppress the proliferation of activated HSCs and promote their death. Ji et al. developed PEG‐PLGA nanoparticles modified with cyclic RGD peptides (cRGD), which are specifically bound to integrin αvβ3, co‐loading GMO and miR‐29b for anti‐fibrotic liver treatment. The accumulation of cRGD‐modified PEG‐PLGA nanoparticles in the activated HSCs significantly reduced the production of collagen I causing considerable anti‐fibrotic effects in the mice model of CCl4‐induced liver fibrosis.^97^ Zhang et al. also developed RGD‐modified hepatitis B core protein nanocages to target quercetin delivery to HSCs. The nanocages accumulated in HSCs via the affinity of the RGD domain to integrin αvβ3 and transformed HSCs into a quiescent phenotype, reducing ECM secretion in vitro and in vivo.^98^ Relaxin (RLX) is a small‐molecule polypeptide with anti‐fibrotic activity. It inhibits the activation and proliferation of fibroblasts and enhances the degradation of collagen fibers. Hu et al. developed lipid‐protamine‐hyaluronic acid (LPH) nanoparticles co‐encapsulating the *relaxin* gene and a miR‐30a‐5p mimic targeted to the sigma‐1 receptor of aHSCs. The LPH nanoparticles delivered a combination therapy involving the crosstalk between macrophages and aHSCs to achieve a synergistic therapeutic effect in mice with fibrosis.^99^ In another study, doxorubicin (DOX) and retinoic acid were encapsulated together into chondroitin sulfate nanomicelles (CS micelles) to target aHSCs in an anti‐fibrotic liver. This in vitro study showed that CS micelles were selectively endocytosed by activated HSCs through CD44 receptors and displayed synergistic therapeutic effects in a CCl4‐induced fibrosis model (Figure 4f).^100^ Luo et al. developed collagenase‐ and silibinin encapsulated nanoparticles coated with chondroitin sulfate to protect the stability of collagenase and target CD44 on aHSCs in vivo. Compared with normal hepatocytes, the nanoparticles accumulated in aHSCs via the chondroitin sulfate targeting function. Furthermore, collagenase broke down the dense collagen stroma and facilitated the penetration of silibinin into aHSCs in vivo, inhibiting fibrosis in mice.^101^ Ribera et al. developed anti‐PDGFRβ‐modified gold nanorods targeted at aHSCs. The nanorods produced thermal ablation under near‐infrared light inducing fibrosis regression in mice with CCl4‐induced fibrosis (Figure 4g).^102^

##### Macrophages as targets

Kupffer cells (KCs) are resident macrophages in the liver that participate in the fibrotic process by altering the cell phenotype. During liver fibrosis, KCs transform from an anti‐fibrotic M1 phenotype into a pro‐fibrotic M2 phenotype.^103^ Specific receptors highly expressed in activated KCs provide a rationale for the delivery of drugs to KCs via ligand‐modified delivery systems that precipitate macrophage repolarization. Kaps et al. developed a nanohydrogel equipped with mannose residues (ManNP), which targeted the delivery of siRNA to M2 polarized macrophages to knockdown the CSF‐1 receptor (CSF‐1R) mRNA in vivo. The siRNA‐loaded ManNPs exhibited good biocompatibility and accumulation in M2‐type macrophages.^104^ In another study, tumor necrosis factor‐stimulated gene 6 (TSG‐6) was found to ameliorate liver fibrosis. Furthermore, Wang et al. developed calcium phosphate nanoparticles (CaP@BSA NPs) for loading TSG‐6 with high loading efficacy. They found that the CaP@BSA NPs improved liver fibrosis by modulating M2 macrophages demonstrating promising anti‐fibrotic effects.^105^

#### 3.3.3 Tissue level

The liver microenvironment exhibits special characteristics during the process of liver fibrogenesis which could be considered as targets against liver fibrosis.^106^ These environmental characteristics could also be considered as sensors for targeted drug delivery and responsive release in the fibrotic microenvironment (Table 3). In one study, a ROS and pH dual‐sensitivity polydatin‐encapsulated micelle (PDPA) was prepared. The micelles fractured in acidic lysosomes, releasing polydatin, which is a promising anti‐fibrotic drug. Moreover, the micelle presented synergistic ROS scavenging activity resulting in the inhibition of liver fibrosis, by preventing hepatocyte apoptosis and the activation of pro‐fibrotic cells in vitro and in vivo.^107^ MMPs degrade ECM proteins and participate in the progression of liver fibrosis, exerting different roles in different stages. MMP‐9 has been found to have an important role in the early stage of liver fibrosis. In another study, Graphene nanostars linked to the PAMAM‐G5 dendrimer were developed to deliver a plasmid encoding for MMP‐9 to cause MM‐9 overexpression and lead to collagen breakdown.^108^ The overexpression of MMP‐targeted therapy reduced the production of collagen in fibrotic tracts markedly reducing hepatic injury.

## 4 CHALLENGES

**FIGURE 5:**
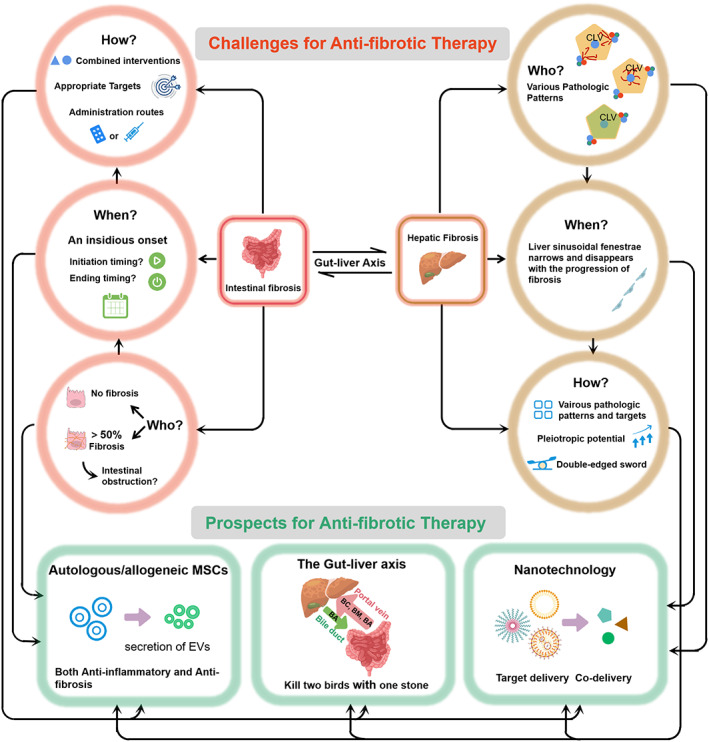
Challenges and opportunities for developing anti‐fibrotic therapeutics for intestinal and hepatic fibrosis. For intestinal fibrosis, the relationship between inflammation and fibrosis should be clarified for patient screening and a personalized therapeutic schedule should be provided. In addition, more than onethird of patients with inflammatory bowel disease experienced nonalcoholic fatty liver disease suggesting a close association between the two diseases, and which is likely influenced by the intestinal microbiome and gut barrier destruction. For hepatic fibrosis, various pathologic patterns pose great difficulty in developing anti‐hepatic fibrosis therapies. The narrowing of the liver sinusoidal fenestrae with fibrosis progression is a challenge currently complicating effective drug delivery. Effective therapies should have multiple targets at different stages. (BC, bacterial composition; BM, bacterial metabolites; BA, bile acid).

Although different anti‐fibrotic strategies at the molecular, cellular, and tissue levels have been discussed in this review, there are still challenges to developing safe, effective, and low‐toxicity anti‐fibrotic therapies (Figure 5).

### 4.1 Intestinal fibrosis

#### 4.1.1 Patient screening for anti‐fibrosis therapies

To date, the relationship between inflammation and fibrosis is still unclear. Local inflammation is known to trigger intestinal fibrosis, however, with the use of biological agents, progress has been achieved regarding anti‐inflammatory treatments. Regardless, there has been no marked decrease in the incidence of intestinal fibrosis. In addition, some inflammatory gut diseases, such as celiac disease and lymphocytic colitis, are not complicated by fibrotic lesions, suggesting that non‐inflammatory factors, such as microorganisms and bacterial products, are also drivers of fibrogenesis.^109^ Inflammation is necessary for fibrosis, but it most likely does not affect fibrotic progression. It is unclear as to which type of patient should receive anti‐fibrotic treatment because not all patients with inflammation develop intestinal fibrosis and obstruction. A method for early identification of patients at high risk for progression to fibrosis is still lacking and is one of the difficulties in anti‐fibrotic therapy.^44^ It is also unclear as to whether patients with complete bowel obstruction should receive anti‐fibrotic therapy. Once fibrous stenosis occurs, the patient must undergo balloon dilatation or strictureplasty to remove the block and it remains unknown whether these interventions impact anti‐fibrotic treatment. Intestinal fibrosis has an insidious onset and its progression is difficult to detect and evaluate until the formation of intestinal stenosis and obstruction. Thus, the exact time of initiation of therapy is unclear. It is crucial to understand the pathophysiology of CD‐associated strictures and predictors of stricturing with accuracy and sensitivity to allow early anti‐fibrotic treatment to slow down the fibrotic process, and possibly even reverse intestinal fibrosis. Additionally, it is difficult to determine when therapy should end due to a lack of accepted clinical trial endpoints and detected responses while undergoing anti‐fibrotic therapy.

#### 4.1.2 The appropriate strategy

As mentioned, previously, it is probable that anti‐inflammatory or anti‐fibrotic treatments alone would not prevent or reverse fibrosis, and should be used in combination with anti‐inflammatory treatments. However, it is unclear as to how “combined interventions” should be developed, including the order and ratio of anti‐inflammatory or anti‐fibrotic drugs. Some studies have shown that the cause of fibrous stenosis is not only the proliferation and deposition of fibrous tissue, but also the proliferation and hypertrophy of intestinal smooth muscle cells (SMCs) within the submucosa and muscularis propria of intestinal stenosis, which also promotes the EMT process in intestinal fibrosis leading to the production of collagen.^110^ The proliferation of SMCs seems to be a unique feature of intestinal stenosis and the underlying cause of increased thickness of the bowel wall stricture, as changes in the SMCs in fibrosis in other organs are not obvious. It is not enough to design and develop a treatment strategy focusing on the process of intestinal fibrosis while ignoring smooth muscle hyperplasia. Treatments targeting SMCs in intestinal fibrosis may be particularly attractive as the strategy would benefit anti‐fibrotic processes by inhibiting EMT, while also directly decreasing the thickness of the bowel wall stricture by preventing smooth muscle proliferation.^22^

The excessive accumulation of ECM components and the proliferation of SMCs increase the density and stiffness of fibrotic lesions, which develops an inaccessible microenvironment and hampers the distribution and accumulation of therapeutic agents.^111^ The effective delivery of drugs into a hypovascularized fibrotic tissue is a substantial challenge. During IV administration, the thickening of the smooth muscle and the increase in fat around the intestinal cavity impair the ability of drugs to reach the mucosal layer where fibroblasts and myofibroblasts are distributed. However, when administering oral preparations, the intestinal mucosal side of the fibrotic site usually exhibits barrier damage, enabling drugs that are targeted to fibroblasts or myofibroblasts to enter the mucosal layer. Consequently, oral or local administration is more suitable for targeting fibroblasts, myofibroblasts, or epithelial interstitial cells and IV administration may be more practical for drug delivery to SMCs which are mainly distributed in the submucosa.

### 4.2 Hepatic fibrosis

#### 4.2.1 Patient screening for anti‐fibrosis therapies

Chronic liver diseases associated with hepatic fibrosis mainly progress through one of three patterns: (1) Chronic viral hepatitis leading to a “post necrotic” pattern, which is the main feature of porto‐central progression, accompanied by sinusoidal arterialization and neoangiogenesis; (2) Chronic alcoholic hepatitis and NASH leading to a “pericentral fibrosis” pattern; or (3) Cholestasis caused by primary biliary cholangitis and primary sclerosing cholangitis, leading to biliary fibrosis.^112^, ^113^ These various patterns most likely differ in their progression to hepatic fibrosis, which is necessary for making individualized clinical decisions on anti‐fibrotic therapy. The key issues are the identification of which patients qualify to be treated more urgently, as well as the recognition of low‐, intermediate‐, or high‐risk advanced fibrosis. Biopsies have been the gold standard for fibrosis assessment, but its limitations are well‐known, including invasiveness, potential complications, sampling errors, and high cost making it non‐attractive to clinicians and patients. Therefore, a precise, non‐invasive, and inexpensive method of screening patients for individualized fibrosis assessment and treatment is still lacking.

Both the free‐drug and the nanoparticle‐based therapies for liver sinusoidal fenestrae easily accumulate in the normal liver following IV administration. Particles of this size can also reach hepatocytes or HSCs, while larger particles are easily phagocytized by KCs.^114^ However, this window of time narrows and disappears with the progression of fibrosis. The size of the delivery system should be designed considering the manner in which it passes through the liver endothelial fenestrae to reach the target cells and achieve maximum concentration. Fibrotic treatment should be administered before the liver sinusoidal fenestrae becomes narrow and disappears. Once the liver sinusoidal fenestrae closes, it is difficult for drugs to reach the hepatocytes or HSCs. Recently, Li et al. utilized a pretreatment with Riociguat which reversed the liver sinusoid capillarization, followed by a targeted delivery of peptide‐nanoparticles encapsulated with the anti‐fibrosis agent JQ1. This strategy highlights the importance of the liver sinusoidal endothelium in anti‐liver fibrosis.^115^

#### 4.2.2 The appropriate strategy

The increased hardness and density of the ECM in fibrosis is the primary barrier in fibrotic tissue to drug delivery and should be considered in the development of delivery systems targeting drugs to fibrotic organs, regardless of the target cell. In addition to the ECM, there are also alterations in the biochemical features of the liver, such as the liver sinusoidal fenestrae, which present challenges to delivery. Furthermore, when targeting liver immune cells for liver fibrosis, the facilitation of nanoparticle endocytosis by immune cells becomes a double‐edged sword. Although macrophages are easily targeted, there is also a probability of them being cleared before they reach liver macrophages. If the targets are missed, then endocytosis by macrophages is reduced. In liver fibrosis, HSCs are located in the space of Disse near the endothelial cells. Such a location makes it difficult to target HSCs, as the delivery system might also be internalized by KCs and liver sinusoidal endothelial cells before reaching HSCs.^116^ Emerging evidence in hepatic fibrosis suggests that different pathologies are involved in the different subtypes of fibrogenic cells. These pathologies may have diverse anti‐fibrotic targets, respond differently to clinical management, and need specific therapeutic drugs. The various liver zones and etiologies may also indicate differences in the rate of liver fibrosis progression, the dynamics of the necroinflammatory infiltration, and the onset and progression of portal hypertension, suggesting barriers to drug delivery systems that remain unclear. Finally, even if the target is specific to one cell type, the pleiotropic potential should not be neglected. The target cell exists in different stages, including activation, proliferation, pro‐fibrogenic factors, and collagen release, but to date, research has targeted only a single mode of action.^117^ Liver fibrogenesis is a complicated process with a complex underlying network. Accordingly, more research is needed to identify the appropriate treatment strategies.

## 5 PROSPECTS

Hepatic fibrosis therapeutics currently under exploration have a broad range of targets based on the mechanism of action (MoA) (Table 1) and include (i) improving metabolic disorders, (ii) preventing the activation and proliferation of HSCs, (iii) promoting the degradation of collagen, and (iv) reducing inflammation and cell death. The complex pathophysiology underlying hepatic fibrosis and its interplay with metabolic syndrome processes is unmistakable, although the link is incompletely understood. It is generally believed that hepatic insulin resistance contributes to the development of hepatic fibrosis, as hepatocytes lose their ability to suppress glucose production in response to insulin while preserving their capacity to generate lipids. In addition, metabolic dysregulation further expands the inflammation cascade, contributing to hepatic fibrosis development.^118^ Targeting the regulation of glycometabolism and lipid metabolism to improve hepatic fibrosis, with treatments including Cotadutide, Elafibranor, Pegbelfermin, Aramchol, Resmetirom, and AZD2693 is a hot research topic. However, Elafibranor and Pegbelfermin have shown no improvement in hepatic fibrosis in phase II and III trials. Hydronidone targeted to HSC proliferation has been shown to reach the primary endpoint with good tolerance in phase II clinical trials. It has also been used during an initial phase III study on hepatitis B with liver fibrosis and is expected to provide the medical demand for hepatitis B liver fibrosis treatment. It is also an important target for the inhibition of excessive collagen deposition in the treatment of hepatic fibrosis. Simtuzumab (SIM) a monoclonal antibody against the enzyme lysyl LOXL‐2, was tested in phase IIb trials. However, this clinical trial has been halted due to its ineffectiveness.^119^ Finally, a chemokine receptor‐2/5 (CCR2/5) antagonist (Cenicriviroc) developed to primarily target inflammation showed good anti‐fibrotic effects in a phase IIb trials (CENTAUR study). In Table 1, the therapeutic in phase clinical trials were based on the specific pathway involved during the hepatic fibrosis process compared to conventional therapeutic approaches. In addition, nanodrugs and gene drugs are gradually being developed and promise to have fewer side effects while requiring lower dosages. However, only a small portion of the phase II trial therapeutics have progressed to phase III which generates doubts on single‐agent therapies. In addition, even when focusing on the same MoAs, trial outcomes for different compounds could be different. We speculate that this is likely due to insufficient concentrations of the therapeutics at the action in vivo requiring multi‐modal synergistic therapy and targeted delivery technology for clinical translation. Besides, several promising therapeutic strategies were summarized as follows (Figure 5).

### 5.1 Mesenchymal stem cells

Mesenchymal stem cells (MSCs) are early undifferentiated cells with the ability of self‐renewal and multi‐directional differentiation. MSC‐based therapies are becoming a hot topic, particularly in autoimmune diseases. Autologous and allogeneic BM‐MSCs have been shown to reduce IBD inflammation in clinical trials.^120^ In addition, clinical studies have demonstrated that autologous or allogeneic adipose‐derived MSCs exert promising therapeutic effects on anal fistulae, closely related to fibrosis.^121^, ^122^ In a recent phase I–II clinical study, local injections of MSCs were found to reverse intestinal fibrosis and resolve intestinal stenosis.^123^ This effect was most likely dependent on their immunoregulatory effects and inhibition of TGF‐β signaling^124^ demonstrating an exciting prospect of simultaneous anti‐inflammatory and anti‐fibrosis effects from MSC therapies. EVs derived from MSCs may also regulate intestinal fibrosis through cargo loading.^125^

MSCs have shown therapeutic effects on liver inflammation and fibrosis through self‐differentiation and EV secretion.^126^ Another study reported that allogeneic BM‐MSCs were able to migrate to the location of liver injury, increase the M2/M1 macrophage ratio, and promote HSC apoptosis revealing that BM‐MSCs are able to relieve liver fibrosis in a CCl4 model.^127^ The cytokines and EVs secreted by MSCs can also alleviate liver fibrosis. Rong et al. showed that BM‐MSC derived EVs were effective in a CCl4 rat model and were able to protect hepatocytes. Other studies have suggested that the anti‐fibrotic effect of EVs derived from MSCs is even greater than that of the MSCs themselves.^128^ The most attractive aspect of MSCs‐based therapy is that there is no need to distinguish which patient type would develop fibrosis because EV‐ and MSC‐based therapies are both anti‐inflammatory and anti‐fibrotic.

### 5.2 Application of nanotechnology

Since its first application in the clinic, there has been great progress in the application of nanotechnology as a drug vehicle. One advantage of nanotechnology is the ability to target specific parts and single cells within an organ for drug delivery, maximizing drug activity while minimizing side effects. The free‐drug possesses promising anti‐fibrotic properties, but its non‐targeted accumulation induces mucosal damage at healthy sites.^129^ There is real opportunity to exploit nanotechnology for targeted drug delivery to intestinal lesions by manipulating the specific pathological differences between normal and fibrotic intestinal tissues. The markers expressed at high levels on fibrotic intestinal tissues, such as α‐SMA, FAP, PDGFR, and FGFR, could be considered as specific targets recognized by ligands modified on the nano‐delivery system. Fibrosis is a complex disorder and there is an urgent need to focus on combination therapies that have different mechanisms of action. Owing to their nanoscale size and structure, nanoparticle delivery systems are especially suitable for the co‐delivery of drugs. Combination therapies using nanoparticles could target a variety of mechanisms, or multiple disease states simultaneously, to provide synergistic therapeutic effects for effective future anti‐fibrotic therapies.

### 5.3 The gut‐liver axis

Several studies found a close association between NAFLD, a major cause of liver fibrosis, and IBD, as up to one‐third of patients with IBD experienced NAFLD.^130^, ^131^ Although the mechanism for the association is unclear, the gut‐liver axis is a link between the intestine and liver, involving a crucial component of the bidirectional relationship. The interaction is created through the portal vein, which transports gut microbial components and metabolites directly to the liver, which then transports bile and metabolites to the intestine.^132^ The interaction between the intestinal flora and the bile acids have been demonstrated to either directly or indirectly affect intestinal and liver diseases.^133^ Bile acids transform into secondary bile acids following metabolization by intestinal flora and are reabsorbed by the liver through intestinal liver circulation. The insufficient flow of bile acids, resulting in deficient luminal levels, can affect the composition of intestinal flora, subsequently inducing abnormalities in the intestinal microbiota. Increased levels of secondary bile acids in the gut lumen can induce alterations in the microbiota, resulting in reduced intestinal FXR signaling, the loss of tight junctions between epithelial cells, thinning of the mucous layer, and a decreased production of anti‐microbial peptides, which contribute to the interaction of pathobionts with mucosal immune systems and lead to intestinal inflammation and fibrosis. When the gut barrier is destroyed, the gut microbial components and metabolites, and even the structure‐altered gut microbiota itself, can easily transfer to the liver promoting liver inflammation and fibrosis. Moreover, the overproduction of secondary bile acids reabsorbed by the liver induces abnormal liver immunity and activates HSCs provoking liver damage and promoting liver fibrosis.^134^, ^135^ The gut‐liver axis should be investigated in intestinal and hepatic diseases. Therapeutics targeting the gut‐liver axis may improve intestinal and hepatic fibrosis through regulation of the intestinal flora and bile acids producing dual benefits.

## 6 CONCLUSIONS

This review comprehensively discussed the pathogenesis, targets, and therapies for intestinal and hepatic fibrosis to highlight the important advances, challenges, and opportunities for anti‐fibrotic therapies. A deeper understanding of the mechanisms of fibrosis, along with more advanced drug delivery technologies, creates an increased need for clinicians, pharmacists, and researchers to further develop integrated strategies encompassing new agents utilizing different delivery systems for effective individualized clinical prospects that treat patients with fibrosis and enhance their quality of life.

## AUTHOR CONTRIBUTIONS

**Xin Li:** Conceptualization (lead); resources (lead); writing – original draft (lead). **Mengli Yu:** Writing – original draft (supporting). **Qingwei Zhao:** Writing – review and editing (supporting). **Yang Yu:** Writing – review and editing (lead).

## CONFLICT OF INTEREST STATEMENT

The authors declare that they have no known competing financial interests or personal relationships that may influence the work reported in this review.

## Data Availability

Data sharing is not applicable to this article as no datasets were generated or analyzed during the current study.
